# Antioxidant properties of potato tubers (*Solanum tuberosum* L.) as a consequence of genetic potential and growing conditions

**DOI:** 10.1371/journal.pone.0222976

**Published:** 2019-09-25

**Authors:** Anna Jadwiga Keutgen, Elżbieta Wszelaczyńska, Jarosław Pobereżny, Agnieszka Przewodowska, Włodzimierz Przewodowski, Dorota Milczarek, Beata Tatarowska, Bogdan Flis, Norbert Keutgen

**Affiliations:** 1 BOKU - University of Natural Resources and Life Sciences, Division of Vegetables and Ornamentals at the Department of Crop Sciences, Vienna, Austria; 2 UTP University of Science and Technology, Department of Microbiology and Food Technology, Bydgoszcz, Poland; 3 Plant Breeding and Acclimatization Institute (IHAR) – National Research Institute, Bonin Research Center, Bonin, Poland; 4 PlantBreeding and Acclimatization Institute (IHAR) - National Research Institute, Młochów Research Center, Młochów, Poland; Institute for Biological Research, SERBIA

## Abstract

The concentrations of the bioactive compounds in potato tubers are determined by both genetic potential and environmental factors. The purpose of the experiment was to determine the influence of organic and integrated production on the expression of the genetic potential with respect to the antioxidant properties of potato tubers and to evaluate its stability under different environmental conditions. This phenotyping was performed on seven new breeding lines (tetraploid clones) and three modern cultivars: Jelly, Satina and Tajfun. The results indicated that production system and location significantly influenced the antioxidant capacity measured by FRAP method. Organic farming and the location Chwałowice were characterized by higher values. Furthermore, anitioxidative capacity measured by FRAP method was correlated with chlorogenic acid content (r = 0.590**) and glutathione fractions, especially with the reduced form (GSH, r = 0.692**). Multidimensional comparative analysis (MCA) proved a better development of antioxidant properties of potato tubers in the organic cultivation system when compared with the integrated. Especially favorable were growing conditions at Boguchwała (organic) and worst at Młochów (integrated). From all investigated varieties, the best antioxidant properties were found in ‘Satina’ and ‘Jelly’. Clones TG-97-403 and 13-VIII-45 developed the weakest health promoting traits.

## Introduction

Potato is the fifth most important staple food crop in the world, which supplies energy and some nutritionally relevant ingredients [1]. The nutritional value of potato tubers is mainly characterized by the presence of essential amino acids (esp. lysine), high contents of starch and dietary fibre as well as a low concentration of fats [2–3]. Potato tubers prepared with no or low fat addition contain also important levels of bioactive compounds and antioxidants [4–8], including phenolic acids (mainly chlorogenic acid, content from 49 to 1400 mg kg^-1^ DM [9–10], ascorbic acid [4, 11–13], and flavonoids (ranging from 200–300 mg kg^-1^ FM), which are phytochemicals helping to reduce the risk of several diseases, such as cardiovascular diseases (CVD) via cholesterol reduction, inflammatory pathways, and cancer [4–18].

A higher consumption of potato tubers may increase the antioxidant level in blood and tissues and acts against oxidative stress, which is responsible for damages of lipids, proteins, enzymes and DNA, resulting in chronic diseases such as cancer or CVD [6, 10, 16, 19–22]. However, there is very limited evidence about antioxidant content in potato tubers as influenced by the genetic potential of cvs realised under different growing conditions, for instance under organic and integrated production. Taking into account the high volume of potato consumption in Europe (per capita dietary intake is about 90 kg year^-1^) potato may serve as an important source of desired phytochemicals in food worldwide [23].

In addition, the above-mentioned secondary metabolites, which are also described as “ecologically active plant compounds”, are essential for maintenance and survival of plant species in ecosystems. These compounds are characterised by physiological and ecological functions and are the result of interactions between the plant and its environment. As a consequence, the improvement of traits (including quality traits) is possible by growing of existing cultivars or lines under conditions that positively influence or enhance expression of the traits. In particular, organic cultivation is expected to positively influence the antioxidative quality of potato tubers [11–12, 24–26]. In addition, tuber quality is correlated with climatic and weather conditions, environment (location) and genotypes [7, 25].

Although antioxidants contents of potato tubers has been the subject of investigation for a long time, knowledge about the influence of interactions between genotype and environment (GxE) on the antioxidative traits in a holistic approach is still limited. The goal of the presented study was to determine the contribution of environmental variation and genetic determination such as represented by the seven new breeding lines (tetraploid clones) and the three modern cultivars (Jelly, Satina and Tajfun). In particular, multi-environmental trials enable to analyse interaction effects and eventually to evaluate the stability of the expression of chosen bioactive compounds. Thus, comprehensive compositional profiles are of interest for the characterization of germplasm for breeding purposes. “Metabolic profiling” is used to screen populations for genotypes with elevated amounts of vitamins and phytonutrients that are constantly produced under a variety of environmental contitions.

## Material and methods

### Plant material

For this study 3 potato (*Solanum tuberosum* L.) cvs (German ‘Jelly’ (‘Jel’) and ‘Satina’ (‘Sat’), Polish ‘Tajfun’ (‘Taj’), all yellow fleshed) and 7 tetraploid breeding lines obtained from a crossing programme performed at the Plant Breeding and Acclimatization Institute–National Research Institute, Młochów Research Center, Poland were used (4 lines are yellow-fleshed and 3 white-fleshed). All used breeding lines are complex hybrids of different *Solanum* species and they were propagated vegetatively.

### Field experiments

**Fig 1:**
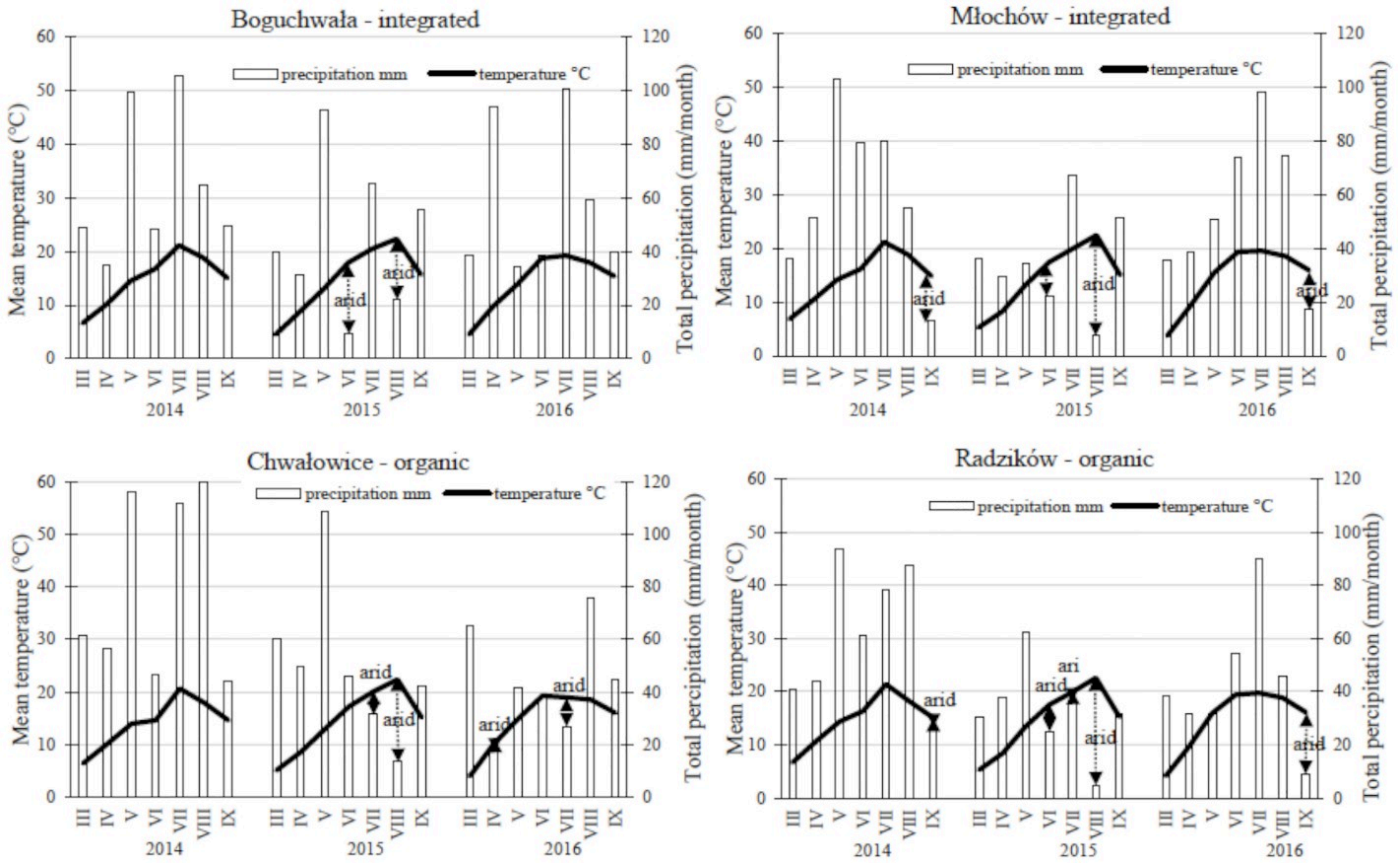
Total monthly precipitation (mm) and monthly average temperature (°C) during the 2014, 2015 and 2016 potato growing seasons at Boguchwała (B), Chwałowice (Ch), Młochów (M) and Radzików (R) as Walter/Lieth-climate diagram.

The experimental design was a randomized complete block with two biological repetitions (blocks) conducted as field experiments in three consecutive years (2014–2016) at four locations in Poland with different soil types (loam and clay soils). At two of them (Boguchwała and Młochów) integrated and at the other (Radzików and Chwałowice) organic farming practices were applied (Table 1). Both of the organic farming sites were certified by the Polish company (AGRO BIO TEST) according to the norm EN 45011 (implemented since 2002, accreditation by the Polish Centre of Accreditation, AC 096). In each of the two blocks potato clones were planted on six hill plots, where the seed tubers’ weight was standardized before planting (700 g per hill plot). In each experimental year tubers were planted at the end of April and harvested after 130 days. Description of the locations and used fertilizers in each experimental year and location are presented in Table 1. Applied fertilizers doses were as recommended by the Polish Ministry of Agriculture published by the Main Inspectorate of Plant Health and Seed Inspection for the integrated production [27]. Weather conditions during the field experiments in 2014–2016 are shown in Fig 1. Crop protection treatments during the vegetation period were applied in accordance with the agrotechnic requirements for potato and the requirements of organic farming (i.e. Novodor FC or pyrethrin as plant extract against Colorado beetle and copper fungicides against late blight disease). Weeding was performed mechanically and manually respectively, when needed. Different locations and production systems were chosen for the investigation of the interaction of genotype vs. environment. An influence of the environment was indicated only in the case, when the mean value of the needed characteristic was better in the given location across all investigated varieties. Detailed description of plant materials and also field trials has already been published by Keutgen et al. [28] and Tatarowska et al. [29].

**Table 1:** Cultivation conditions (location, soil type, fertilization) for potato production in different locations and years.

Location	Geographic coordinates	Type of soil	Type of production	Year	Doses of fertilizers kg ha^-1^
Boguchwała	49° 58′ 59″ N 21° 56′ 23″ E	Cambisols		2014	120 N, 60 P_2_O_5_, 180 K_2_O + 15t composted manure + foliar fertilization Adob Cu, Adob Mn, Adob S, Basfoliar 36 Extra
			integrated	2015	120 N, 60 P_2_O_5_, 180 K_2_O + foliar fertilization Basfoliar 36 Extra, Basfoliar 12-4-6+S+amino
				2016	120 N, 60 P_2_O_5_, 180 K_2_O + foliar fertilization Basfoliar 36 Extra, Basfoliar 12-4-6+S+amino
Młochów	52° 3′ 0″ N 20° 46′ 7″ E	Podzols		2014	
			integrated	2015	green manures (white mustard), 90 N, 110 P_2_O_5_, 180 K_2_O
				2016	
Chwałowice	51° 10′ 56″ N 21° 18′ 17″ E	Cambisols		2014	25 t/ha composted manure
			organic	2015	
				2016	
Radzików	52°13’38"N 20°36’55 "E	Phaeozem		2014	25 t/ha composted manure
			organic	2015	
				2016	

### Sample preparation

Immediately after harvest the tubers were sent to UTP Bydgoszcz, Poland, for further preparation and analyses (within 1 day). After transportation and 3 days of incubation of potato tubers in the laboratory storage at 10 °C and 80% r.h. the tubers were cut and well mixed. A representative sample was frozen in liquid nitrogen and stored at least at -18 °C for further investigations. In frozen tubers the contents of ascorbic acid were measured. For other analyses the frozen material was freeze-dried (Alpha model 1–4 LDplus, Donserv, Warsaw, Poland). Immediately after the drying process, the dry matter content was determined. Dried potato tubers were milled to obtain a fine powder (particle size 0.3–0.5 mm mesh) using an Ultra-Centrifuge Retsch mill ZM 100 (Retsch, Germany). Milled samples were stored in darkness in air-tide bags in desiccators until further analyses.

### Chemical analyses

The antioxidants analyses comprised: the contents of total phenolics, chlorogenic acid (main phenolic compound in potato tubers), total flavonoids, reduced (GSH) and total glutathione, ascorbic and citric acid as well as oxidative and antioxidative potential of collected tubers (four replications). All spectrophotometric measurements were performed with Shimadzu UV-1800, Kyoto, Japan.

Preparation and measurement of L-ascorbic acid (LAA) were performed as described by Albrecht [30] and the amounts of LAA were calculated per kilogram fresh mass.

The contents of glutathione (reduced GSH, oxidized GSSG and total glutathione) were determined according to the Enzyme-Recycling-Method of Anderson as described and modified by Keutgen and Pawelzik [31] based on the reaction of glutathione with 5,5’-dithio-bis(2-nitrobenzoic acid) DTNB in the presence of glutathione-reductase and NADPH using the Glutathione Assay Kit (Sigma–Aldrich, Germany), Cat. No CS0260.

The concentration of total phenolic compounds was determined spectrophotometrically using the colorimetric assay with Folin-Ciocalteu’s phenol reagent according to Singleton and Rossi [32] with methanol as extraction agent (0.5 g of freeze-dried material/10 ml solution triple extracted as described by Keutgen and Pawelzik [31]). The measurement was performed at 736 nm and the results were expressed in mg gallic acid equivalents (GAE) kg^-1^ dry mass.

Chlorogenic acid was determined spectrophotometrically according to the sodium nitrite procedure described by Griffiths et al. [33] at a wavelength of 510 nm. For comparison, a standard curve of chlorogenic acid in the range from 50 to 400 mg L^-1^ was used.

For the measurement of citric acid, the argentometric method of Paczinok [34] was used. Total flavonoids were determined colorimetrically in ethanol extracts of potato tubers according to Eberhardt et al. [35]. The absorbances at 510 nm were measured against the prepared blank in comparison with standards prepared with known (+)-catechin concentrations. The results were expressed in mg of catechin equivalents per kilogram dry mass.

The antioxidant capacity was evaluated spectrophotometrically at 593 nm using the Ferric ion Reducing Antioxidant Power (FRAP) assay as previously described by Keutgen and Pawelzik [31].

### Statistic analyses

All statistical analyses were performed with IBM SPSS Statistics version 24.0 for Windows. After testing the data for normal distribution and variance homogeneity, the mean values obtained in the different groups were compared by One-Way ANOVA, at a significance level of 0.05 by Tukey b test or Wilcoxon-Mann-Whitney-Test (U-test) depending on the results of the Levene tests. Furthermore, a multifactorial ANOVA with year as a cofactor at a significance level of 0.05 was also applied. Results are presented as mean values plus standard deviation. Correlation coefficients were determined between bioactive compounds and antioxidant capacity using the Pearson coefficient at P ≤ 0.01 when results were normally distributed.

The evaluation of the tested genotypes under the conditions of integrated and ecological cultivation as well as of the various cultivation sites was achieved using multidimensional comparative analysis (MCA), which deals with the comparison of multi-feature objects [28]. Using MCA, the genotypes are hierarchized from the point of view of antioxidant properties and oxidative state of potato tubers. The method of linear ordering of the investigated objects has been applied. At the same time, a matrix was created for a specific moment of time (a particular growing season), which presents the implementation of individual features (diagnostic variables) for each genotype-environment combination (objects). Standardization was applied in the form of a series of transformations of this matrix, which allowed obtaining values of features devoid of designation and on this basis to create a single synthetic variable representing a complex phenomenon. This variable is referred to as a synthetic measure of the investigated genotype, which is the result of the MCA. MCA values are the main criterion for organizing the examined objects and their ranking. In the process of normalization, the considered diagnostic features have been assigned a specific meaning for the assessment of objects. So-called stimulants are those features, whose larger values are more desirable with respect to the object’s assessment. Destimulants are features, where smaller values are preferred. In the presented studies, the selected genotypes were rated using the following diagnostic features: X1 –chlorogenic acid g kg^-1^ DM (destimulant–main cause of darkening processes in potato tuber), X2 –total phenolics g kg^-1^ DM (destimulant–discolourations and negative taste changes), X3 –FRAP mmol Fe^2+^ kg^-1^ DM (stimulant–antioxidative capacity as a result of all antioxidants present), X4 –total flavonoids g kg^-1^ DM (stimulant—antioxidant), X5 –ascorbic acid mg kg^-1^ FM (stimulant—antioxidant), X6 –citric acid g kg^-1^ FM (stimulant–antioxidant), X7 –total glutathione mmol kg^-1^ DM (stimulant -antioxidant), X8 –reduced glutathione GSH mmol kg^-1^ DM (stimulant—antioxidant), X9 –oxidized glutathione GSSG mmol kg^-1^ DM (destimulant–used, inactive form of GSH).

## Results

At all locations temperature developed rather similar during the three years of field experiments (Table 1 and Fig 1). Substantial differences between locations could not be detected (mean temperature for all locations and years was 14.6 °C ± 0.16 °C). Significant differences occurred for precipitation. In Fig 1, the arrows marked with the dotted lines describe arid conditions on the field for this time interval, the columns area above the temperature line describe humid conditions, which are suitable for the development of *Phytophthora infestans*. Especially in 2014, conditions were favourable for the development of late blights disease due to the long wet weather periods during cultivation. In 2015, several arid periods occurred at all investigated locations. Conditions were most humid at Chwałowice with the mean sum of annual precipitation during the vegetation period being 406.1 mm ± 132.0 mm, followed by Boguchwała (390.3 mm ± 68.2 mm), Młochów (350.6 mm ± 90.4 mm), and Radzików (320.7 mm ± 103.0 mm). Generally, a high variation in precipitation at each location was observed among the years (Fig 1). The so manifested different production conditions are favorable for the assessment of the stability of quality traits, especially in the new breeding lines.

The results of this study indicated that potato tubers grown under two different production systems irrespective of location and variety or clone showed significant differences in the content of total phenolics, total flavonoids, ascorbic acid, and citric acid (Tables 2–4). Integrated production with better growing conditions favoured the synthesis of citric acid of about 4.9% (Tables 2 and 4) in comparison with organic farming. Under conditions of moderate stress as in the case of organic farming the production of phenolic compounds (Tables 2 and 4) was 15%, of flavonoids 45% and of ascorbic acid 8.5% higher than under integrated production. In consequence, tubers from the organic system were characterised by a 13.9% higher antioxidant capacity measured by FRAP method and reached a value of 16.3±6.67 mmol Fe^2+^ kg^-1^ DM (Tables 3 and 4).

**Table 2:** Content of antioxidants of potato tubers depending on cultivation system and used genotype in three years of field experiments.

Property	Total Phenolics (g kg^-1^ DM)		Total Flavonoids (g kg^-1^ DM)		Citric Acid (g kg^-1^ FM)		Oxidized Glutathione (mol kg^-1^ DM)	
Genotype/ *prod*. *system*	*integrated*	*organic*	*integrated*	*organic*	*integrated*	*organic*	*integrated*	*organic*
clone 13-VIII-10	2.99±0.77^abcd^	3.24±0.62^abcd^	0.57±0.14^bc^	0.12±0.03^b^	0.15±0.04^ab^	0.76±0.33^abc^	4.58±0.25^ab^	4.50±0.25^abc^
clone 13-VIII-27	2.72±0.64^cd^	3.27±0.47^abcd^	0.46±0.19^c^	0.15±0.02^ab^	0.16±0.02^a^	0.84±0.19^abc^	4.28±0.62^a-f^	3.84±0.71^c-f^
TG-97-403	2.64±0.35^d^	3.18±0.60^abcd^	0.55±0.24^bc^	0.15±0.02^ab^	0.16±0.02^ab^	0.94±0.07^abc^	4.37±0.42^a-e^	4.56±0.45^a-f^
clone 13-VIII-45	3.11±0.52^abcd^	3.48±0.66^abcd^	0.70±0.38^abc^	0.16±0.02^ab^	0.16±0.03^ab^	1.05±0.59^ab^	4.42±0.49^a-e^	4.12±0.61^a-f^
clone 13-VIII-49	2.85±0.60^bcd^	3.12±0.61^abcd^	0.48±0.21^c^	0.13±0.01^ab^	0.14±0.01^ab^	0.74±0.40^abc^	4.48±0.39^a-d^	4.30±0.48^a-f^
clone 13-VIII-50	3.32±0.98^abcd^	3.65±0.61^ab^	0.63±0.27^abc^	0.16±0.05^ab^	0.15±0.02^ab^	0.96±0.13^abc^	4.67±0.38^a^	4.64±0.39^a^
clone 13-VIII-60	2.81±0.35^bcd^	3.68±0.84^ab^	0.55±0.18^bc^	0.17±0.02^a^	0.16±0.02^ab^	1.08±0.62^a^	3.78±0.45^ef^	3.92±0.65^b-f^
Jelly	3.22±0.79^abcd^	3.59±0.84^abc^	0.76±0.63^abc^	0.16±0.03^ab^	0.17±0.03^a^	0.73±0.27^abc^	4.14±0.48^a-f^	3.80±0.50^d-f^
Satina	3.17±0.51^abcd^	3.80±0.44^a^	0.58±0.12^bc^	0.17±0.03^a^	0.15±0.03^ab^	0.77±0.26^abc^	4.05±0.49^a-f^	3.65±0.50^f^
Tajfun	3.13±0.50^abcd^	3.63±0.53^abc^	0.70±0.25^abc^	0.15±0.02^ab^	0.17±0.04^a^	0.86±0.36^abc^	4.29±0.39^a-f^	4.12±0.39^a-f^
Single effects	A = 0.002	B = 0.000	A = 0.113	A = 0.000	B = 0.233	B = 0.000	A = 0.000	B = 0.001
Interactions	AxB = 0.795		AxB = 0.011		AxB = 0.054		AxB = 0.540	
Co-factor	Year = 0.000		Year = 0.000		Year = 0.000		Year = 0.000	

Different letters after the mean values indicate significant differences by Tukey b test at p≤0.05 within the given property among the investigated genotypes and production systems. A multifactorial ANOVA was used in order to test for single effects of genotype and production systems as well as for interactions. In addition, the significance of the co-factor ‘year of experiment’ was tested; A: cv. / clone, B: production system.

**Table 3:** Content of antioxidants of potato tubers depending on cultivation system and used genotype in three years of field experiments.

Property	Total Glutathione (mol kg^-1^ DM)		Reduced Glutathione (mol kg^-1^ DM)		Oxidized Glutathione (mol kg^-1^ DM)		Antioxidant Capacity (mmol Fe^2+^ kg^-1^ DM)	
Genotype/ *prod*. *system*	*integrated*	*organic*	*integrated*	*organic*	*integrated*	*organic*	*integrated*	*organic*
clone 13-VIII-10	0.27±0.10^a^	0.30±0.07^a^	0.15±0.08^a^	0.15±0.08^a^	0.12±0.03^b^	0.15±0.04^ab^	12.2±5.5^a^	13.9±5.5^a^
clone 13-VIII-27	0.33±0.10^a^	0.33±0.10^a^	0.19±0.09^a^	0.16±0.05^a^	0.15±0.02^ab^	0.16±0.02^a^	14.0±6.5^a^	14.9±7.0^a^
TG-97-403	0.32±0.09^a^	0.32±0.05^a^	0.17±0.07^a^	0.16±0.05^a^	0.15±0.02^ab^	0.16±0.02^ab^	13.0±5.0^a^	14.7±6.6^a^
clone 13-VIII-45	0.34±0.10^a^	0.34±0.06^a^	0.18±0.09^a^	0.18±0.05^a^	0.16±0.02^ab^	0.16±0.03^ab^	16.2±6.4^a^	19.4±8.5^a^
clone 13-VIII-49	0.29±0.07^a^	0.30±0.05^a^	0.16±0.06^a^	0.16±0.05^a^	0.13±0.01^ab^	0.14±0.01^ab^	12.8±4.5^a^	13.2±4.2^a^
clone 13-VIII-50	0.33±0.15^a^	0.33±0.12^a^	0.17±0.10^a^	0.18±0.10^a^	0.16±0.05^ab^	0.15±0.02^ab^	16.5±7.2^a^	17.3±6.0^a^
clone 13-VIII-60	0.36±0.07^a^	0.34±0.07^a^	0.19±0.06^a^	0.19±0.08^a^	0.17±0.02^a^	0.16±0.02^ab^	13.9±3.7^a^	20.1±8.1^a^
Jelly	0.35±0.11^a^	0.35±0.09^a^	0.19±0.09^a^	0.19±0.08_a_	0.16±0.03^ab^	0.17±0.03^a^	12.5±4.8^a^	13.7±4.9^a^
Satina	0.38±0.11^a^	0.33±0.10^a^	0.21±0.08^a^	0.18±0.08^a^	0.17±0.03^a^	0.15±0.03^ab^	15.4±5.0^a^	18.1±6.4^a^
Tajfun	0.36±0.14^a^	0.38±0.15^a^	0.21±0.14^a^	0.21±0.12^a^	0.15±0.02^ab^	0.17±0.04a	16.6±5.9^a^	18.2±6.4^a^
Single effects	A = 0.000	B = 0.9950	A = 0.003	B = 0.522	A = 0.000	B = 0.233	A = 0.000	B = 0.000
Interactions	AxB = 0.766		AxB = 0.874		AxB = 0.054		AxB = 0.399	
Co-factor	Year = 0.000		Year = 0.000		Year = 0.000		Year = 0.000	

Different letters after the mean values indicate significant differences by Tukey b test at p≤0.05 within the given property among the investigated genotypes and production systems. A multifactorial ANOVA was used in order to test for single effects of genotype and production systems as well as for interactions. In addition, the significance of the co-factor ‘year of experiment’ was tested; A: cv. / clone, B: production system.

**Table 4:** Content of antioxidants of potato tubers depending on cultivation system in 2014–2016 in field experiments.

Property	Phen	Flavo	Vit. C	Citric A	FRAP
Prod. System					
Integrated	3.00±0.64^b^	0.60±0.30^b^	25.8±8.73^b^	4.31±0.50^a^	14.3±5.57^b^
Organic	3.46±0.65^a^	0.87±0.39^a^	28.0±6.22^a^	4.10±0.58^b^	16.3±6.67^a^

Different letters indicate significant differences by t-tests at p≤0.05 within the given property (n = 240).

Phen—total phenolics (g kg^-1^ DM), Flavo—total flavonoids (g kg^-1^ DM), Vit. C—ascorbic acid

(mg kg^-1^ FM), Citric A—citric acid (g kg^-1^ FM), FRAP–antioxidant capacity (mmol Fe^2+^ kg^-1^ DM)

The co-factor year had a significant influence on almost all antioxidant properties of potato tubers (Tables 2, 3 and 5). The development of total flavonoids, total glutathione, and both glutathione fractions was the highest in the third experimental year, especially in comparison with the first experimental year. Phenolic compounds were found at the highest concentration of 3.60 ±0.66 g kg^-1^ DM in the second year of investigations (Table 5). The highest amount of ascorbic acid with 30.8±5.37 mg kg^-1^ FM and antioxdiative capacity with 18.9±4.06 mmol Fe^2+^ kg^-1^ DM were observed in the first year (more humid experimental year) (Table 5).

**Table 5:** Content of antioxidants of different potato tubers depending on the year of experiment irrespective of the production system and genotype.

Property	Chlor A	Phen	Flavo	Vit. C	Citric A	Glut	GSH	GSSG	FRAP
Prod. system									
1. Year	1.20±0.48^b^	2.81±0.45^c^	0.54±0.30^b^	30.8±5.37^a^	4.21±0.38^b^	0.27±0.04^b^	0.12±0.03^b^	0.15±0.02^b^	18.9±4.06^a^
2. Year	2.29±0.96^a^	3.60±0.66^a^	0.83±0.39^a^	23.9±7.57^b^	3.90±0.62^c^	0.28±0.05^b^	0.13±0.03^b^	0.15±0.02^b^	18.6±4.93^a^
3. Year	0.60±0.29^c^	3.28±0.68^b^	0.88±0.35^a^	26.0±8.05^b^	4.52±0.43^a^	0.45±0.07^a^	0.28±0.05^a^	0.17±0.03^a^	8.5±2.11^b^

Different letters indicate significant differences by t-tests at p≤0.05 within the given property (n = 160).

Chlor A—chlorogenic acid (g kg^-1^ DM), Phen—total phenolics (g kg^-1^ DM), Flavo—total flavonoids (g kg^-1^ DM), Vit. C—ascorbic acid (mg kg^-1^ FM), Citric A—citric acid (g kg^-1^ FM), Glut—total glutathione (mol kg^-1^ DM), GSH–reduced glutathione (mol kg^-1^ DM), GSSG–oxidized glutathione (mol kg^-1^ DM), FRAP–antioxidant capacity (mmol Fe^2+^ kg^-1^ DM)

A more complicated picture was found in the case of the tested cultivars and clones (Tables 2, 3 and 6). Statistically significant differences irrespective of the growing conditions were found in the case of total phenolic compounds, citric acid, total and oxidized glutathione. In the case of total phenolics cv. Satina, here used as a standard cv. due to its high palatability and acceptance of consumers, was characterized by a significantly higher concentration (3.48±0.57 g kg^-1^ DM) than clone TG-97-403 (2.91±0.55 g kg^-1^ DM). The amount of citric acid was higher in tubers of clone 13-VIII-50 (4.66±0.38 g kg^-1^ FM) compared to clones 13-VIII-27 (4.06±0.69 g kg^-1^ FM), TG-97-403 (4.27±0.44 g kg^-1^ FM), 13-VIII-45 (4.27±0.56 g kg^-1^ FM), and 13-VIII-60 (3.85±0.56 g kg^-1^ FM) as well as to cvs Jelly (3.97±0.51 g kg^-1^ FM), Satina (3.85±0.53 g kg^-1^ FM), and Tajfun (4.21±0.39 g kg^-1^ FM). A significantly higher content of total glutathione was determined for tubers of ‘Jelly’ (0.35±0.08 mol kg^-1^ DM) and clone 13-VIII-60 (0.35±0.10 mol kg^-1^ DM) compared to clone 13-VIII-10 (0.28±0.09 mol kg^-1^ DM). Cv. Satina (0.16±0.03 in mol kg^-1^ DM) and clone 13-VIII-60 (0.16±0.02 in mol kg^-1^ DM) were characterized by significantly higher amounts of oxidized glutathione by contrast to the clones 13-VIII-10 (0.14±0.04 in mol kg^-1^ DM) and 13-VIII-49 (0.13±0.01 mol kg^-1^ DM). However, the detected differences did not influenced significantly the antioxidative capacity of tubers measured as FRAP.

**Table 6:** Antioxidant properties of 10 potato cultivars and clones independent of growing conditions over three years experiment.

Property	Chlor A	Phen	Flavo	Vit. C	Citric A	Glut	GSH	GSSG	FRAP
Genotype									
clone 13-VIII-10	1.12±0.92^a^	3.12±0.70^ab^	0.87±0.52^a^	26.0±7.17^a^	4.54±0.25^ab^	0.28±0.09^b^	0.15±0.08^a^	0.14±0.04^bc^	17.8±7.54^a^
clone 13-VIII-27	1.12±0.69^a^	3.00±0.61^ab^	0.81±0.52^a^	27.8±6.24^a^	4.06±0.69^bcd^	0.33±0.10^ab^	0.17±0.08^a^	0.15±0.03^abc^	17.4±6.07^a^
TG-97-403	1.24±0.82^a^	2.91±0.55^b^	0.80±0.37^a^	26.1±5.48^a^	4.27±0.44^bcd^	0.32±0.07^ab^	0.17±0.06^a^	0.15±0.02^ab^	17.0±6.90^a^
clone 13-VIII-45	1.43±0.97^a^	3.30±0.61^ab^	0.78±0.31^a^	30.2±9.39^a^	4.27±0.56^bcd^	0.33±0.08^ab^	0.18±0.07^a^	0.16±0.03^ab^	16.9±6.44^a^
clone 13-VIII-49	1.13±0.66^a^	2.98±0.61^ab^	0.75±0.47^a^	28.9±9.11^a^	4.39±0.44^abc^	0.30±0.06^ab^	0.16±0.05^a^	0.13±0.01^c^	16.8±5.77^a^
clone 13-VIII-50	1.70±1.11^a^	3.48±0.81^ab^	0.75±0.34^a^	26.5±7.88^a^	4.66±0.38^a^	0.33±0.13^ab^	0.18±0.09^a^	0.15±0.04^abc^	14.5±6.62^a^
clone 13-VIII-60	1.50±1.09^a^	3.25±0.77^ab^	0.68±0.22^a^	27.2±8.56^a^	3.85±0.56^d^	0.35±0.08^a^	0.18±0.07^a^	0.16±0.02^a^	13.9±5.74^a^
Jelly	1.39±0.96a	3.40±0.82^ab^	0.67±0.27^a^	25.0±7.45^a^	3.97±0.51^cd^	0.35±0.10^a^	0.19±0.08^a^	0.16±0.03^ab^	13.1±4.78^a^
Satina	1.69±1.20^a^	3.48±0.57^a^	0.65±0.27^a^	23.2±6.63^a^	3.85±0.53^d^	0.36±0.10^ab^	0.19±0.08^a^	0.16±0.03^a^	13.0±5.41^a^
Tajfun	1.33±0.83^a^	3.38±0.56^ab^	0.61±0.26^a^	28.1±6.62^a^	4.21±0.39^cd^	0.37±0.14^ab^	0.21±0.12^a^	0.16±0.03^ab^	13.0±4.26^a^

Different letters indicate significant differences by U-test at p≤0.05 within the given property (n = 48).

Chlor A—chlorogenic acid (g kg^-1^ DM), Phen—total phenolics (g kg^-1^ DM), Flavo—total flavonoids (g kg^-1^ DM), Vit. C—ascorbic acid (mg kg^-1^ FM), Citric A—citric acid (g kg^-1^ FM), Glut—total glutathione (mol kg^-1^ DM), GSH–reduced glutathione (mol kg^-1^ DM), GSSG–oxidized glutathione (mol kg^-1^ DM), FRAP–antioxidant capacity (mmol Fe^2+^ kg^-1^ DM

A multidimensional comparative analysis (MCA) was performed in order to evaluate the dependency of the antioxidants pool on the production system, location and investigated cultivars and clones (Table 7). The MCA revealed that the organic production system favoured the synthesis of antioxidant properties compared to integrated cultivation. Investigated cultivars and clones showed also an excellent performance in the range from 0.699 to 1.000 rel. units, where the standard cv. Satina and cv. Jelly showed the highest possible synthetic measures due to the higher development of antioxidant properties in potato tubers (Table 7). The lowest performance was found at the clones TG-97-403 (0.699) and 13-VIII-45 (0.701), which cannot be recommended for the further breeding program with the target of high health promoting compounds.

**Table 7:** Synthetic values (rel. units) after multidimensional comparative analysis MCA of antioxidant properties of potato tubers based on cultivation system, location and genotype. The highest value reflects the best combination of chosen properties (no. of considered properties = 10).

Prod. System	MDA_system_	Location	MDA_location_	Genotype	MDA_genotype_
Integrated	0.699	Młochów	0.772	TG-97-403	0.699
Organic	0.856	Radzików	0.792	clone 13-VIII-45	0.701
		Chwałowice	0.898	clone 13-VIII-27	0.736
		Boguchwała	0.926	clone 13-VIII-10	0.787
				Tajfun	0.802
				clone 13-VIII-49	0.804
				clone 13-VIII-50	0.831
				clone 13-VIII-60	0.831
				Jelly	0.907
				Satina	1.000

## Discussion

In the presented research, it has been confirmed that the choice of the genetic potential, growing site and production system significantly influenced the antioxidant properties of potato tubers [4–5]. The differences between organic and integrated production due to the distinct agricultural practices resulting in a variable mineral nutrition and application of chemical/artificial pesticides in organic farming, may modify quality, taste and the amounts of pesticide residues. Under organic farming a higher content of dry matter, mineral nutrients, amino acids, and chosen antioxidants as well as lower nitrate content in potato tuber were reported [11–12, 24–26, 36–37]. In the present study, under conditions of moderate stress as usual in the case of organic farming, the production of phenolic compounds, flavonoids and ascorbic acid was significantly higher than in the integrated production system. These findings are not in line with Ezekiel et al. [3], who did not find any differences in phenolic contents in tubers grown in organic and integrated farming systems. By contrast, Lombardo et al. [11] and Grudzińska et al. [4] found higher amounts of total phenolics in potato tubers when cultivated organically. The higher concentration of total phenolics may represent the result of a stronger ‘pathogenic pressure’ or of a lower nitrogen availability and increased contents of phenolic defence compounds as a strategy of increased resistance to pests and diseases [11]. In the present experiment the observed increase of total phenolics was due to the rise of phenolic compounds other than chlorogenic acid, which is in contrast to Hamouz et al. [24]. In addition, the growing site did influence the content of phenolic compounds and chlorogenic acid; the lowest amounts of both were found at Młochów. To a lesser extent not only the phenolic compounds but also chlorgenic acid, were influenced by variety. The highest amounts of phenolic compounds were found in cv. Satina (3.48±0.57 g kg ^-1^ DM), which is a result of the genetic ability of this cultivar to accumulate phenolics. This result confirms the findings of Gugała et al. [38]. Flavonoids showed a similar development such as phenolic compounds and their function is mainly combined with the role as a yellow pigment in the tuber. However, differences among cultivars with respect to flavonoid development were not observed despite different flesh colours of the selected cultivars. Worthy of note, flavanols are present in small amounts in potato tubers. They develop a strong antioxidant activity and are associated with several potential health properties [6]. Together with other constituents such as proteins or polysaccharides they contribute to the sensoric properties of the commodity (flavour, colour and astringency) [39]. Moreover, they oxidize quite easily and may cause unwelcome discolorations. The contents of ascorbic acid in the presented experiments was on average 25.8±8.73 and 28.0±6.22 mg kg^-1^ FM for integrated and organic system, respectively, and organic farming resulted in significantly higher concentrations, which is in contrast to Grudzińska et al. [4] and Smith-Spangler et al. [13]. The discrepancy may be explained by a limited availability of nitrogen in the organic production systems studied here and, thus, smaller amounts of above ground mass, for the buildup of which photosynthates could have been used. As a consequence, additional carbohydrates could have been partitioned towards the synthesis of ascorbic acid. Tubers from the locality Chwałowice were characterised by the highest ascorbic acid content, possibly due to adequate environmental conditions, in addition to the best agricultural practice. Differences among the tested cultivars with respect to ascorbic acid were not found, which is in contrast to Lombardo et al. [11, 37] and Leo et al. [40], who reported cultivar effects. Moreover, they confirmed that environmental conditions are an important determinant of antioxidant accumulation in potato tubers. In addition to ascorbic acid, also glutathione (especially reduced glutathione GSH) contributes to the detoxification of reactive oxygen species in plant cells as part of the Foyer-Halliwell-Asada cycle. With respect to the glutathione fractions, differences between the production systems were not found. This indicates that the occurring moderate stress under organic farming promoted only the synthesis of ascorbic acid. Comparison of the growing sites showed a higher content of total glutathione in tubers from Radzików compared to Boguchwała and Chwałowice due to higher contents in both fractions, GSH and GSSG. There are also differences among varieties: Cv. Jelly and clone 13-VIII-60 were characterized by higher amounts of glutathione than clone 13-VIII-27. This may implicate a genetic predisposition. Further differences were found in the content of citric acid, which inhibits browning processes in potato tubers. Higher concentrations are reported for tubers grown in integrated production, especially in those produced at Młochów. These results may indicate that citric acid acts as an antioxidant in potato tuber in the first line to resist abiotic stress and discoloration processes. The largest differences in the quality were found for citric acid content in the investigated varieties, which implicates different susceptibilities to browning processes in the tubers [22]. The highest amounts were detected in clones 13-VIII-50 and 13-VIII-10 (less susceptible to browning processes) in comparison to clone 13-VIII-60 in addition to the cvs Jelly, Satina, and Tajfun (more susceptible to browning processes). As a combined value of antioxidant properties the antioxidant activity measured as FRAP in potato tubers grown organically was significantly higher than that of tubers grown in an integrated manner; the differences between Chwałowice and Młochów were especially distinct. The present result confirms Grudzińska et al. [4], although Lombardo et al. [11] could not find any influence of the cultivation system on the level of antioxidant activity as a result of joint contribution of several antioxidant compounds (including ascorbic acid and some phenolics). In addition, the present study could not detect statistically significant differences among the investigated clones and cultivars; the Ferric ion reducing antioxidant power ranged from 13.0–17.8 mmol Fe^2+^ kg^-1^ DM. Moreover, the FRAP value was positively correlated with higher contents of chlorogenic acid, of total phenolics, the percentage of chlorogenic acid to total phenolics, and the percentage of oxidized glutathione GSSG to total glutathione. The strongest correlations were found in the cases of chlorogenic acid (r = 0.590**) and the percentage of chlorogenic acid to total phenolics (r = 0.565**). The antioxidative capacity FRAP of tubers was negatively influenced by an increase of the content of total phenolics other than chlorogenic acid, citric acid, total glutathione as well as both of its fractions (GSH and GSSG) and the increase of the coefficient GSH to GSSG as well as the percentage of GSH to total glutathione. The strongest negative relationships were observed for GSH (r = -0.692**), %GSH (r = -0.674**), total glutathione (r = -0.652**) and the coefficient GSH/GSSG (r = -0.629**). Thus, it can be concluded that the increase of the antioxidant properties of potato tubers are in the first line combined with the fractions of glutathione and with the content of chlorogenic acid.

In the case of so many different clones and cultivars grown under a large variety of different environmental conditions and based on so many tuber quality properties it is difficult to elaborate a clear statement, which cultivar and production system is better with respect to a certain locality (affected by soil type and local climate). However, it is a common problem to characterize a cultivar by several properties, because such an evaluation has a multidimensional character. The knowledge about the relationship of several traits is crucial for a clear statement, resulting in the challenge to determine, which combination is most suitable [29]. In order to solve this problem, it is important to standardize the different properties, which allows an effective comparison. Usually, such kind of multidimensional comparative analysis (MCA) method is used in economy, e.g. for the assessment of stocks or companies. It could be proved by MCA that organic farming generally favoured the synthesis of antioxidant compounds in potato tubers, confirming Lombardo et al. [11, 37] and Grudzińska et al. [4]. However, the differences though significant are not large. Two groups of growing sites could be distinguished, where Boguchwała (integrated) and Chwałowice (organic) were more suitable for potato production for environmental reasons independent of the production system. Of special interest for future breeding is the ranking of the well-established cultivars and new bred clones, which should be characterized by higher contents of health promoting compounds. Irrespective of the locality, production system and the year of experiment, the best cultivars were the middle early ‘Satina’ and the middle-late ‘Jelly’, both from German breeders. Both are characterized by low requirements for N-fertilization (about 100 kg N ha^-1^ [41]) and an average susceptibility to abiotic stress. Surprisingly cv. Tajfun was rated worse than the other cvs, although it is recommended for organic farming due to little environmental requirements and resistance to pests [41]. The main difference among the three cultivars was the higher fertilizer requirements of cv. Tajfun (recommended N dose of 120 kg ha^-1^ [41]). The new bred clones TG-97-403 and 13-VIII-45 were characterized by a considerably worse production of antioxidants. These clones are still under examination for their suitability for different production systems, especially for organic farming. In the first line, they were developed for higher contents of carotenoids [29]. This does not necessary result at the same time in a stable or appropriate acclimatization to different growing conditions and generally high production of antioxidants. The tetraploid breeding line TG-97-403 is characterized by a contribution of *Solanum phureja* in its pedigree (about 15.6%), which was chosen for a higher resistance to *Phytophthora infestans* and for higher carotenoids contents in tubers [29]. Clone 13-VIII-45 had as an exception a single pollen parent and a seed parent, both with high contents of carotenoids in their tubers (other clones had seed parents of low content). This pronounced characteristic may negatively influence the general production of different antioxidants. However, this growth response shows that the theoretically high genetic potential is not necessarily appropriately realized under the different realistic growing conditions. From the new bred lines the clones 13-VIII-49, 13-VIII-50 and 13-VIII-60 are rated as very promising with respect to their contents of antioxidative compounds. Clones TG-97-403, 13-VIII-45, 13-VIII-27, and 13-VIII-10 are not satisfying for health promoting purposes.

## Conclusions

The conducted research on the influence of the production system, locality and potato cultivars on the content of particular antioxidant compounds revealed that potatoes can contribute to a higher intake of antioxidants in the human diet due to the quantity and regularity of potato consumption [14, 42]. Especially organic farming caused a significant increase of the antioxidants pool and antioxidative activity of potato tubers at least in the case of the investigated varieties. However, an up to 50% lower yield should be taken into account. The results confirmed a main influence of the production system, the locality, and the year of experiment, the latter reflecting an impact of the weather conditions. The choice of the potato variety played a secondary role. However, the well-established cvs Satina and Jelly produced higher concentrations of the desired health promoting compounds and can be recommended for both integrated and organic production. In conclusion, this study is a relevant contribution to the breeding programs with the target of enhancing the antioxidant properties of potato tubers and implicate the use of new and improved clones such as 13-VIII-49, 13-VIII-50 and 13-VIII-60 for further work, especially for organic farming. The application of a multidimensional comparative analysis (MCA) allowed an effective evaluation of the influence of chosen factors on many antioxidant properties in a more sophisticated way by building a synthetic value that enables a comparison in order to draw a general conclusion. Future studies shall continue to validate the chosen, promising clones for their sensory properties and tolerance to diseases such as late blight. Finally, the effect of other factors, such as storage and processing ability should be considered.
